# *PMEPA1* Gene Isoforms: A Potential Biomarker and Therapeutic Target in Prostate Cancer

**DOI:** 10.3390/biom10091221

**Published:** 2020-08-22

**Authors:** Shashwat Sharad, Albert Dobi, Shiv Srivastava, Alagarsamy Srinivasan, Hua Li

**Affiliations:** 1 Center for Prostate Disease Research, John P. Murtha Cancer Center Research Program, Department of Surgery, Uniformed Services University of the Health Sciences and Walter Reed National Military Medical Center, Bethesda, MD 20817, USA; adobi@cpdr.org (A.D.); shsr629@gmail.com (S.S.); alagarsamy.srinivasan@gmail.com (A.S.); 2 Henry M Jackson Foundation for the Advancement of Military Medicine, Bethesda, MD 20817, USA

**Keywords:** *PMEPA1*, TMEPAI, isoform, androgen receptor, TGF-β, NEDD4

## Abstract

The identification of prostate transmembrane protein androgen induced 1 (*PMEPA1*), an androgen responsive gene, came initially from the studies of androgen regulatory gene networks in prostate cancer. It was soon followed by the documentation of the expression and functional analysis of transmembrane prostate androgen-induced protein (*TMEPAI*)/*PMEPA1* in other solid tumors including renal, colon, breast, lung, and ovarian cancers. Further elucidation of *PMEPA1* gene expression and sequence analysis revealed the presence of five isoforms with distinct extracellular domains (isoforms *a*, *b*, *c*, *d*, and *e*). Notably, the predicted amino acid sequences of PMEPA1 isoforms show differences at the N-termini, a conserved membrane spanning and cytoplasmic domains. *PMEPA1* serves as an essential regulator of multiple signaling pathways including androgen and TGF-β signaling in solid tumors. Structure-function studies indicate that specific motifs present in the cytoplasmic domain (PY, SIM, SH3, and WW binding domains) are utilized to mediate isoform-specific functions through interactions with other proteins. The understanding of the “division of labor” paradigm exhibited by *PMEPA1* isoforms further expands our knowledge of gene’s multiple functions in tumorigenesis. In this review, we aim to summarize the most recent advances in understanding of *PMEPA1* isoform-specific functions and their associations with prostate cancer progression, highlighting the potentials as biomarker and therapeutic target in prostate cancer.

## 1. Introduction

Prostate transmembrane protein androgen induced 1 (*PMEPA1*) is classified as a type 1b transmembrane protein with luminal, membrane spanning and cytoplasmic domains [1]. The predicted amino acid sequences of *PMEPA1* protein from diverse species showed that the gene is highly conserved in evolution. *PMEPA1* is a multifunctional protein and plays very critical roles in prostate tumorigenesis [1]. Reduced *PMEPA1* (isoform *-b*) expression leads to up-regulated AR protein, activation of AR signaling, and subsequent accelerated prostate cancer cell growth [2,3,4]. As a TGF-β responsive gene, *PMEPA1* (isoform *-a* and -*d*) also inhibits TGF-β signaling via a negative feedback loop. Additionally, it has been shown that *PMEPA1* gene drive tumorigenesis through interference with other signaling cascades such as EGF, Wnt, mutated p53, and Hippo signaling [1,5,6,7,8,9,10,11,12]. The elucidation of *PMEPA1/STAG1/TMEPAI* gene sequences at the mRNA level reveals multiple isoforms. Currently, 5 *PMEPA1* isoforms (*a*, *b*, *c*, *d*, and *e*) are identified with distinct expression and function pattern in prostate cancer [1,11]. This review highlights the pleiotropic nature of *PMEPA1* gene isoforms in the context of molecular structures and cancer biology, especially androgen and TGF-β signaling.

## 2. Discovery of *PMEPA1* Gene Isoforms

**Figure 1:**
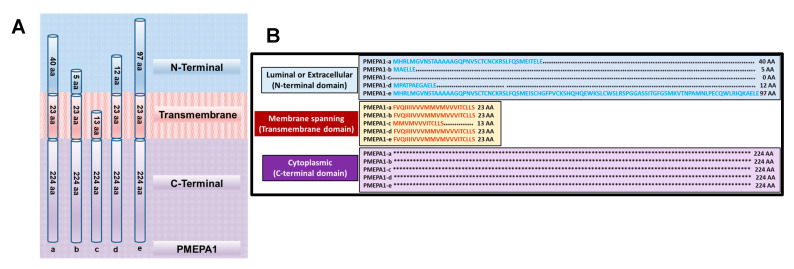
Schematic representation of the predicted domain structure of the isoforms of prostate transmembrane protein androgen induced 1 (*PMEPA1*) protein. (**A**) Three function domains were predicted with a type 1b membrane protein: N-terminal (luminal/extracellular) (blue), membrane spanning (red) and cytoplasmic (purple). (**B**) Alignment of the predicted domain and amino acid (aa) sequences of PMEPA1 isoforms.

The *PMEPA1* gene was first discovered as androgen inducible gene in hormone responsive LNCaP cells. The Northern blotting assay showed two bands corresponding to 5.0 kb and 2.7 kb. The analysis of a cDNA clone containing 1141 bp sequences, derived from the *PMEPA1* gene, revealed an open reading frame (ORF) of 759 nucleotides coding for a protein of 252 amino acids with a predicted molecular mass of 27.8 kDa. The same clone was further spanned over two genomic clones mapped to chromosome 20q13.2–q13.33 and chromosome 20q13.31–q13.33 [5]. In renal cell carcinoma, the characterization of the full-length cDNA clone encompassing *PEMPA1* gene revealed an ORF coding for a protein of 287 amino acids designated as solid tumor associated gene 1 (*STAG1*) [6]. The amino acid sequence homology analysis and chromosome localization suggested this transcript as an isoform of *PMEPA1* gene, which was designated as *STAG1 (PMEPA1* isoform *-a*) [3,5]. Subsequently, additional three isoforms of *PMEPA1* gene were reported with ORF coding for 237 amino acids (PMEPA1-c) in colon and breast cancer [7,13], 259 amino acids (PMEPA1-d) in lung cancer [14] and 344 amino acids (PMEPA1-e) in prostate cancer [1]. Interestingly, it is noted that *PMEPA1* isoforms were also reported as other aliases with different designations before in literature, *PMEPA1-a* (as *STAG1*, *TMEPAI*, *N4wbp4*, *PMEPA1-β*, *PMEPA1A*, *PMEPA1a*), *PMEPA1-b* (as *PMEPA1, PMEPA1-α, PMEPA1B, PMEPA1variant A, TMEPA1, PMEPA1b*), *PMEPA1-c* (as *PMEPA1 variant B*, *PMEPA1C*), and *PMEPA1-d* (as *TMEPAI D*) [6,7,13,14,15,16,17,18,19,20,21]. The protein coded by all the five *PMEPA1* isoforms share high homology at the C-termini but exhibit striking variations at the N-termini (Figure 1).

## 3. Structure and Biochemical Features of *PMEPA1* Isoforms

The analysis of predicted structural features of PMEPA1 protein contains the following three domains: (i) Luminal (N-terminal domain), (ii) Membrane spanning (Transmembrane domain), and (iii) Cytoplasmic. N-terminal domain shows length polymorphisms among the isoforms [11]. The transmembrane and cytoplasmic domains show highly conservative amino acid sequences. PMEPA1-a isoform has 40 amino acids in the luminal domain, 224 amino acids in the intracytoplasmic domain and 23 amino acids in the membrane spanning domain. The isoforms b, d, and e have luminal domain of 5, 12, and 97 amino acids respectively. All these four isoforms share the conserved 23 amino acids in membrane spanning domain and 224 amino acids in the cytoplasmic domain. Only PMEPA1-c isoform contains a truncated membrane spanning domain of 13 amino acids and lacks N-terminus domain. The predicted structural features of proteins coded by PMEPA1 isoforms are schematically shown in Figure 1 with luminal domain (blue), transmembrane domain (red) and cytoplasmic domain (purple). Three distinct motifs are present in the cytoplasmic domain: (i) residues PPPY (158–161) and PPTY (229–232), similar to WW consensus binding sequences, (ii) PPNR (186–189) residues, implicated in binding to Smad, and (iii) PPRP (112–115), PTYP (135–138), and PCPP (205–208), similar to PXXP consensus for binding SH3 domains [6,14,22]. The phosphorylation site analysis of PMEPA1 protein uncovered five putative casein kinase II (amino acids 37, 151, 190, 231, and 254) and four potential protein kinase C (amino acids 68, 116, 182, and 199) phosphorylation sites, along with three consensus motifs for N-linked glycosylation (N-X-T/S) at N8, N19, and N188 and two potential N-myristoylation sites (amino acids 6 and 213) [6]. However, these phosphorylation of PMEPA1 protein are yet to be experimentally confirmed. Of the three potential N-linked glycosylation sites noted in PMEPA1-a isoform, only one site is present in the cytoplasmic domain. Similarly, motifs such as YPYL (residues 138–141), YSEV (residues 232–235) and a di-lucine motif (residues 255–256) located in the cytoplasmic domain of PMEPA1-a protein (Table 1) are also noted in other transmembrane proteins. Despite the high conservation of amino acids noted between PMEPA1 isoforms, various gene polymorphisms at individual residue level have also been documented [23]. The locations of amino acids indicating PMEPA1 protein polymorphism are listed in Table 2. The amino acid sequence changes at these residues are mainly of non-conservative nature and involve residues 3, 75, 128, 179, 220, and 228 [6,13]. Notably, such changes are away from regions encompassing membrane spanning domain, WW domain and PY motifs. The functional significance of these residues is not clear and needs further investigation.

## 4. Evolution and Architecture of *PMEPA1* Gene and Its Isoforms

**Figure 2:**
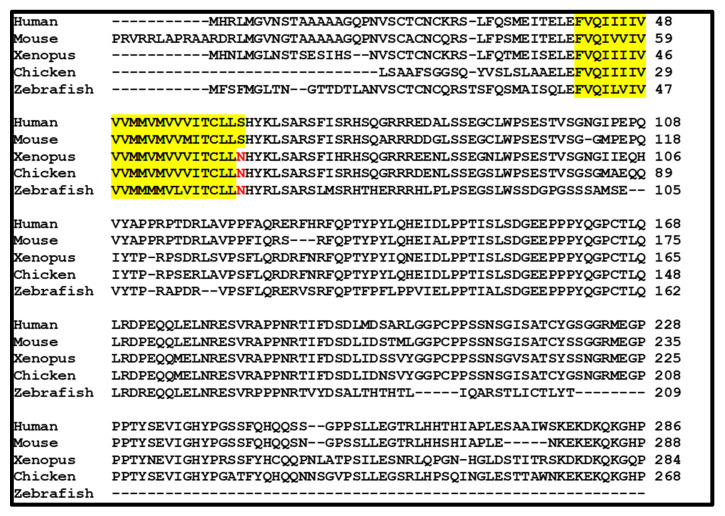
The alignment of the predicted amino acid sequence of *PMEPA1* derived from different species. The sequences corresponding to the membrane spanning domain are indicated (yellow). Overall, the homology between different species is high in distinct regions of the protein. *PMEPA1* from different species also show variation at the N-terminus like the pattern noted among the isoforms of human origin. Additionally, there are distinct differences in the region close to the C-termini of the protein.

The *PMEPA1* gene is conserved in diverse species including mouse, rat, chicken, and xenopus. The alignment of amino acid sequences of PMEPA1 from human, mouse, xenopus, chicken, and zebrafish is shown in Figure 2. Although PMEPA1 proteins from different species show minor variations, the high level of amino acid sequence identity was detected in both N- and C-terminal regions including all conserved functional domains. Due to its highly conservative sequences within species, it is tempting to speculate that *PMEPA1* may play an important role in the mechanisms controlling cell growth and development.

**Figure 3:**
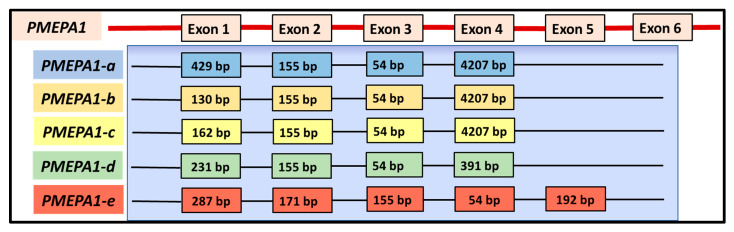
*PMEPA1* isoforms exon-intron structure: Genome schematic representation indicating the structures of *PMEPA1* isoforms and respective intron-exon corresponding to *PMEPA1* gene [1].

The *PMEPA1* mRNA sequence is comprised within the genomic clones designated as RP5-1059L7 (AL21913), RP4-718J7 (A1035541) and RP5-1007E6 (AL161943), which are located in chromosome 20q13.2–q13.33 between D20S183 and D20S173 micro satellite marker 1, 3, and 4. *PMEPA1* gene is approximately 62 kb in length and contains six exons (ranges from 29 bp to 4207 bp) and five introns (ranges from 434 bp to 51,626 bp) [1]. The *PMEPA1* isoforms *a*, *b*, and *d* contain four exons and three introns, whereas isoform c contains three exons and two introns. Isoform *e* contains four exons and three introns. The predicted respective exon-intron structure of *PMEPA1* isoforms (*a–e*) are represented in Figure 3 [1]. The RNA Seq data analysis of PMEPA1-e isoform further reveals an additional unique 57 amino acids from residue 37 to 93, which may be likely due to intron retention between exon 2 and 4 maintaining the open reading frame [1].

## 5. Expression of *PMEPA1* Isoforms

*PMEPA1* transcript was detected predominantly in hormone-dependent organs such as prostate and ovary with Northern blot assay. The in-situ hybridization further showed that *PMEPA1* isoform (*-a* and *-b*) was mainly expressed in benign prostate glandular epithelial cells [5,6]. It was noted that overexpression of *PMEPA1* (isoform *-a*) was detected in multiple solid tumors including breast cancer, colon cancer, renal cancer, stomach cancer, rectal adenocarcinomas, pancreatic endometrial and prostatic adenocarcinomas [6,7,13]. On the other hand, a decrease or loss of *PMEPA1-b* expression was also detected in 64.5% of prostate cancer patients with Quantitative-PCR assay in matched benign and malignant prostate tissue [2]. The discrepancy of *PMEPA1* expression status in various tumor context was likely the result of detection of different *PMEPA1* gene isoforms and different hormone signaling mechanism involved.

Assessments of relative expression of each isoform indicated that *PMEPA1-a* was most abundant followed by *PMEPA1-b* and *PMEPA1-c* in prostate cancer cell lines and human prostate tumor tissues which are defined as major isoforms [1,11]. *PMEPA1-d* and *PMEPA1-e* isoforms are marked as minor isoforms with lower transcript levels detected [1]. The mRNA levels of five *PMEPA1* isoforms was analyzed in various prostate cell lines including AR-positive and androgen-sensitive LNCaP, VCaP, LAPC4 cells, androgen independent CWR22v1, C4-2B cells, AR negative and TGF-β signaling positive DU-145, PC-3 and benign prostate epithelial BPH-1, PrEC cells [1]. The transcripts of *PMEPA1-a*, *PMEPA1-d*, and *PMEPA1-c* were detected in both AR positive and negative cell lines. In contrast, the expression levels of *PMEPA1-b* and *PMEPA1-e* isoforms were found only in AR positive cell lines such as LNCaP, VCaP, LAPC4, CWR22v1 and C4-2B cells but not in AR negative PC-3 and DU-145 cells [1,11]. All of these finding implicated that these isoforms are more associated with androgen or TGF-β regulation in prostate cancer context and their expression levels detected by Q-PCR were showed in Table 3.

The *PMEPA1* isoform-based analysis revealed that the mRNA level of *PMEPA1-b* was higher than *PMEPA1-a* in normal prostate epithelial cells [11], highlighting the functional role of *PMEPA1-b* in homeostasis of androgen regulation. More importantly, the expression of *PMEPA1-b* isoform was decreased in prostate tumors [2,11,25]. In contrast, the expression of *PMEPA1-a* isoform was not altered significantly in prostate tumor compare to benign prostate. The expression of *PMEPA1* gene have been reported to be regulated by androgen, TGF-β, and EGF [26]. Our previous study showed that *PMEPA1* (isoform *-b*) is a direct transcriptional target of AR, and androgen treatment induced the expression of *PMEPA1* (isoform *-b*). ChIP assay further revealed androgen-dependent binding of AR to androgen response elements of *PMEPA1* gene (isoform *-b*) in RC-165N/hTERT and LNCaP cells [27]. *PMEPA1* is also identified as an early response gene to TGF-β [13]. In normal murine mammary gland cells, TGF-β has been shown to induce PMEPA1 expression in a Smad-independent manner [17,28]. High expression of *PMEPA1* (isoform *-a*) was detected in estrogen receptor/progesterone receptor–negative and human epidermal growth factor receptor-2–negative breast cancer cell lines and primary breast cancers and this expression is increased by TGF-β treatment [29]. Further, basal and TGF-β–induced expression of *PMEPA1* (isoform *-a*) is inhibited by TGF-β receptor antagonist SB431542 and overexpression of Smad7. Our recent studies clearly categorize five *PMEPA1* isoforms into the following two subgroups: (1) androgen-responsive *PMEPA1-b* and *PMEPA-e*, whose expressions were induced by linear dosages of androgen and ectopic AR in androgen responsive prostate cancer cells; and (2) TGF-β-responsive *PMEPA1-a*, *PMEPA1-c*, and *PMEPA-d* which were up-regulated by TGF-β treatment and over-expression of TGF-β receptor I [1,11]. Such different expression patterns highly suggest *PMEPA1* isoforms assume distinct biological functions in the context of androgen or TGF-β signaling in prostate cells. Additionally, increased expression of *PMEPA1* in response to decitabine treatment of prostate cancer cells highly suggested that gene methylation contributes to the repression of *PMEPA1* [25]. Expectedly, the high frequency of methylation of *PMEPA1* gene was detected in prostate cancer frozen tissue samples which is also highly correlated to reduced expression of *PMEPA1*.

## 6. *PMEPA1-b* Isoform Inhibits AR Signaling

Ectopic *PMEPA1* (isoform *-b*) degraded AR protein and decreased expression of AR responsive gene PSA in prostate cancer cells. In contrast, loss of *PMEPA1* (isoform *-b*) led to enhanced expression level of AR and subsequent AR signaling activation. Our previous study had revealed that loss or reduced expression of *PMEPA1* (isoform *-b*) was significantly associated with higher PSA level at diagnosis in prostate cancer patients [2,25]. *PMEPA1* (isoform *-b*) protein was found to regulate AR protein stability via a negatively regulated feedback loop. As androgen inducible protein, *PMEPA1* (isoform *-b*) mediated AR protein degradation in proteasome dependent way by recruiting E3 ubiquitin ligase NEDD4, which was also independent of MDM2/p53 signaling pathway [22]. *PMEPA1* (isoform *-b*) protein had been shown to interacts with WW domains of NEDD4 protein directly [2]. The alterations in tyrosine residue in PPPY (158–161 amino acids) and PPTY (229–232 amino acids) in *PMEPA1* (isoform *-b*) protein interrupted such interaction to NEDD4 protein and subsequent AR ubiquitination and degradation. Furthermore, loss of *PMEPA1*(isoform *-b*) disrupted the binding between AR and NEDD4 [4]. Our recent research further highlighted *PMEPA1-b* isoform rather than other *PMEPA1* isoforms to bind to AR protein directly and facilitate the AR protein ubiquitination in a NEDD4-dependent way [11]. All these findings suggest that *PMEPA1-b* protein mediates AR protein degradation via providing the docking platform for binding of AR and ubiquitin ligase NEDD4. In addition, NEDD4 was also found to be androgen responsive in hormone responsive prostate cancer cells. NEDD4 was reported to control the stability of RNA Polymerase II and degradation of PTEN protein [30,31]. Therefore, silencing of *PMEPA1-b* resulted in enhanced PTEN protein degradation via activated AR signaling and subsequent enhanced androgen inducible NEDD4 protein [4].

Similarly, silencing of *PMEPA1* gene (isoform *-b*) resulted in and accelerated growth of LNCaP cells in vitro by activation of androgen signaling. Expectedly, *PMEPA1* (isoform *-b*) depletion facilitated the xenograft growth of LNCaP cells in nude mice in male hormone dependent manner. Moreover, it was observed that LNCaP cells derived xenograft rapidly developed androgen independent growth in castrated nude mice [4]. Notably, knockdown of *PMEPA1* (isoform *-b*) in androgen responsive cells (LNCaP and VCaP cells) led to the development of resistance to AR inhibitors such as enzalutamide and bicalutamide [4]. Our analysis further identified only *PMEPA1-b* specifically suppressing AR signaling and subsequently inhibiting the cell growth, cell plating efficiency and colony formation of androgen responsive LNCaP cells [11]. Taken together with the hormone independent growth of *PMEPA1*-depleted xenograft of LNCaP cells in nude mice, it is implied that the *PMEPA-b* isoform might contribute to progression into castration resistance stage via manipulating AR signaling in prostate cancer cells. Intriguingly, it was also noted that *PMEPA1-b* also inhibits the proliferation of AR signaling negative PC-3 and DU-145 cells. The mechanism of such inhibitory impacts was still not clear.

## 7. *PMEPA1* Isoforms (*a* and *d*) Inhibit TGF-β Signaling

*PMEPA1* (isoform *-a*) was also reported to control the duration and intensity of TGF-β/Smad signaling through a negative feedback loop [14,24]. As a TGF-β responsive gene, *PMEPA1* (isoform *-a*) was reported to antagonize TGF-β signaling in the following five ways: (1) interfering with TGF-β type I receptor-induced R-Smad phosphorylation; (2) directly interacting with R-Smads via a Smad interaction motif (SIM domain), PPNR (178–181 amino acids), and competing with Smad anchor for receptor activation—theMH2 domain in Smad2 is shown to be enough for PMEPA1(isoform *-a*) binding [32]; (3) binding to Smad2/3 and further preventing phosphorylation of the TGF-β receptor kinase complex [24] (interestingly, membrane anchoring was not required for mediating such inhibitory effect of *PMEPA1* protein on TGF-β signaling); (4) directly decreasing Smad3 nuclear translocation in the absence and presence of TGF-β, and MYC partially contributed to *PMEPA1*-induced transcription suppression [18]; and (5) promoting lysosome degradation of TGF-β receptor [33]. As expected, *PMEPA1* (isoform *-a*) over-expression resulted in a decrease of expression levels of TGF-β target genes such as plaminogen activator inhibitor (PAI-1), JunB, cdk inhibitors.

Different from cell growth inhibitory function in context of androgen responsive prostate cancer cells, the *PMEPA1* gene (isoform *-a* and -*d*) was revealed to promote the growth of non-prostate solid tumors including breast and lung cancers via interrupting TGF-β signaling. Loss of *PMEPA1* (isoform *-a*) reduced TGF-β–induced growth and motility in breast cancer cells, and inhibited breast tumor xenograft growth with increased expression of PTEN and moderate phosphorylation of Akt [17,29]. Knockout of *PMEPA1* (isoform *-a*) also led to reduction of xenograft growth and lung metastasis of breast cancer cells, which was associated with downregulation of vascular endothelial growth factor alpha (VEGFA) and interleukin-8 (IL8) [24]. Silencing of *PMEPA1*(isoform -*d*) in lung cancer Calu3 and NCI-H2 cells dramatically inhibited the cell growth in vitro and subcutaneous tumor formation in vivo through enhanced Smad2 phosphorylation in TGF-β dependent way [14]. *PMEPA1* (isoform *-a* and -*d*) was also reported to promote the proliferation of A549 lung cancer cells [34]. More importantly, TGF-β induced expression of *PMEAP1* (isoform -*d*) in lung adenocarcimoma tissue facilitated the epithelial-mesenchymal transition (EMT) by modulating the ROS and IRS-1 signaling pathways [20]. In colorectal cancer cells, *PMEPA1* gene induced EMT process via activating the bone morphogenetic proteins (BMP) signaling of TGF-β signaling network, and subsequently leading to accelerated proliferation and metastasis of tumor cell cells [26]. In androgen independent PC-3 prostate cancer cells, depletion of *PMEPA1* gene (isoform *-a*) facilitated the bone metastasis of xenograft in nude mice through activating TGF-β signaling and subsequent up-regulation of TGF-β responsive pro-metastatic genes including *Il11*, *Adam13* and *Mmp9* [19]. Previous study had detected the expression of 2 *PMEPA1* isoforms (*PMEPA1-a* and *PMEPA1-b*) in PC-3 cells [18]. Additionally, inhibition of *PMEPA1* gene (isoform *-a*) caused the suppression of the growth of AR negative RWPE1 and PC-3 cells. In contrast, ectopic PMEPA1 increased cell proliferation, decreased p21 expression and up-regulation expression of c-MYC. Further analysis showed the function of *PMEPA1* (isoform *-a*) was mainly dependent on its binding to Smad2/3 and Smad3/4 [18].

It has been clarified that three *PMEPA1* isforms (*PMEPA1-a*, *-b* and *-c*) inhibited TGF-β signaling by binding Smad family members (Smad 2 and 3) proteins directly [19]. Recently, our study clarified that *PMEPA1-a* and *PMEPA1-d* were *PMEPA1* isoforms promoting the cell growth, cell plating efficiency and colony forming capacity in soft agar of AR negative prostate cancer cells. Knockdown of TGF-β I receptor dramatically reversed such growth promoting effects. Similarly, *PMEAP1* isoforms (*a* and *d*) significantly up-regulated the transcript levels of TGF-β responsive genes including *NEDD-9*, *THBS1* as well as enhance SMAD luciferase activity [1], which further highlighting the key biological functions of *PMEPA1-a* and *PMEPA1-d* isoforms be tightly associated with TGF-β signaling. Additionally, PMEPA1-c isoform with truncated N-terminal extracellular and transmembrane domains was not found to interfere with the growth of prostate cancer cells, implicating that these two domains be essential to maintain cell growth regulating effects of *PMEPA1* isoforms in prostate cancer cells. In summary, differential regulation of TGF-β signaling by *PMEPA1-a* and *PMEPA1-d* isoforms contributes androgen independent, TGF-β controlled cell growth.

## 8. *PMEPA1* Isoforms: Potential New Biomarkers and Hormone Therapies

Our study had shown that reduced expression level of *PMEPA1-b* was highly associated with bone metastasis in cohort of 120 matched benign and malignant prostate cancer frozen samples [11]. On the contrary, enhanced transcript level of *PMEPA1-a* correlated to biochemical reoccurrence (BCR) in same cohort. The study utilizing PC-3 cells also confirmed the anti-metastasis function of *PMEPA1* gene in prostate cancer cells via inhibiting TGF-β signaling. TGF-β inducible PMEPA1-*a* and PMEPA1-*d* were detected in PC-3 cells. Hence, *PMEPA1* isoforms (*a*, *b* and *d*) were implicated to be involved in mediating metastasis of prostate cancer. The Cancer Genome Atlas (TCGA) data analysis further validated the biomarker potential of *PMEPA1* isoforms for prostate tumor progressions [1,11]. Lower *PMEPA1-b* expression and a higher ratio of *PMEPA1-a* versus *PMEPA-b* were all correlated to higher Gleason scores and lower progression free survival rate in the cohort composing of 499 prostate cancer patients [11]. The higher expression ratios of *PMEAP1-b* versus -*d* or *-e* strongly associated with enhanced Gleason score [1]. Taken together, *PMEPA1* isoforms might function as biomarkers for monitoring disease progression and aggressive clinical outcome via representing surrogate for status of androgen and TGF-β signaling in prostate cancer.

**Figure 4:**
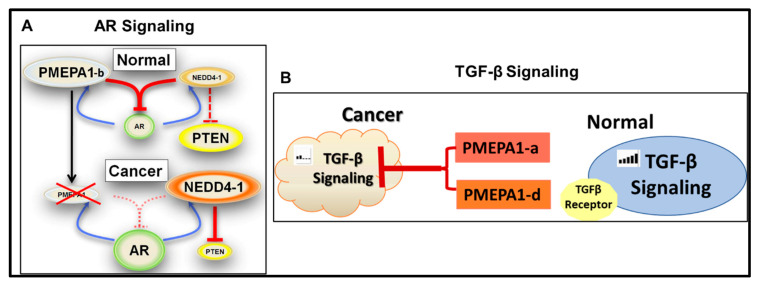
(**A**) Loss or decreased *PMEPA1-b* in prostate cancer cells leads to elevated AR and reduced PTEN: In prostate cancer silencing of *PMEPA1-b*, enhanced NEDD4 protein through activated AR signaling facilitated PTEN degradation, which suggested NEDD4 mediated PTEN degradation was *PMEPA1-b* independent in contrast to AR degradation. The solid line represents the known interaction and the dotted lines indicates the known mechanism. (**B**) *PMEPA1* isoforms (-*a* and -*d*) inhibit TGF-β signaling: Differential regulation of TGF-β signaling by *PMEPA1-a* and *PMEPA1-d* isoforms contributes to androgen independent, TGF-β controlled cell growth. All these findings suggest *PMEPA1* gene utilize the specific isoforms in order to navigate and drive cancer progression.

Dysfunction of androgen and TGF-β signaling played essential roles in prostate cancer progression into hormone treatment resistance stage. The switch of androgen to TGF-β signaling, enhanced AR protein level particularly AR variants including ARv7 all contributed to incidence of castration status. The combination of inhibitors of androgen and TGF-β had been shown to significantly decrease prostate tumor burden in DNTGFRII mice compared to single agent [35]. *PMEPA1* gene manipulated biological activity and stability of both androgen and TGF-β signaling via its isoform specific functions in prostate tumorigenesis and disease progression. Furthermore, PMEPA1-b isoform functioned as a strong degrader of wild-type AR protein. It is worthwhile to further investigate its ability to degrade AR variants such as ARv7 and Arv567 which highly associated to castration resistance and metastasis of prostate cancer [36]. The roles of PMEPA1 gene isoforms in the regulation of androgen and TGF-β signaling switch during the development of hormone therapy resistance also warranted further clarification. Taken together, the multifunction of PMEAP1 isoforms over AR/TGF-β signaling implied a new venture of anti-prostate cancer hormone inhibitory therapy. PMEPA1 isoforms effectively interacted with other cellular proteins partners for mediating the functions (Table 4). Substitution of residues in key functional motifs abrogated the downregulation of AR and the inhibitory effect of *PMEPA1* on TGF-β signaling (Table 5). It was suggested that PMEPA1 is likely an adaptor molecule similar to suppressor gene DAB2, involved in multiple receptors mediated signaling pathways. The dissection of biological function domains of PMEPA1 isoforms associated with AR and TGF-beta signaling potentially lead to novel anti-cancer therapeutics development (Figure 4A,B).

## 9. Future Research Directions

The multi-functional features of *PMEPA1* gene were mainly attributed to its specific isoforms in the various cellular signaling contexts, such as AR and TGF-β signaling. The specific response element sequences to androgen or TGF-β treatments have been localized in the promoters of *PMEPA1* gene, highlighting the expression of *PMEPA1* gene is inducible to both androgen and TGF-β. However, the switching mechanisms of these promoters to navigate the gene expression to different functional isoforms need to be further clarified in tumorigenesis, which is important to elucidate the prostate cancer progression into castration resistance and metastatic stages. Additionally, PMEPA1-b isoform has been proven to be a key modulator of androgen signaling through protein degradation mechanism, which shed the light on the development of new androgen inhibition strategy. The novel small molecule drug harboring the key functional domains of PMEPA1-b isoform might target AR variants associated with disease progression. To further explore the development of new anti-AR variants, the direct protein-to-protein binding and subsequent protein degradation mediated by interactome complex of PEMAP1-b/AR variants/NEDD4 needs to be fully understood. It is also imperative to investigate the associations of protein levels of AR variants, PMEPA1-b and NEDD4 in prostate cancer tissue samples, which further required to generate the PMEPA1 isoform specific antibodies to distinguish the amino acid sequence differences within N-terminus. Although our research has revealed the strong correlation of abnormal transcript levels of PEMPA1 isoforms (*PMEPA1-a* and *PMEPA1-b*) to aggressive prostate cancer outcome, the larger cohort still needs to validate these findings. In addition, the studies of functional associations of *PMEPA1* isoforms to androgen/TGF-β responsive gene cluster during disease progression are also warranted to further establishment of *PMEPA1* gene isoforms as complementary biomarkers of clinical outcome by surrogating androgen/TGF-β signaling status. The promoter methylation of *PMEPA1* gene partially contributed to lower expression of *PMEPA1-b* isoform in prostate cancer cells, which may be helpfully to explain the irregular activation of androgen signaling in prostate tumorigenesis. It was also noted significant more methylation of PMEPA1 gene promoter was detected in Caucasian American (CA) than African American (AA) patients [25]. Whether epigenetic events contribute to abnormal expression levels of other *PMEPA1* isoforms as well as the health disparity in such events is also required to be further clarified. In addition to AR and TGF-β signaling, it is worthwhile to explore the connection between *PMEPA1* isoforms and other major pro-oncogenic signaling/molecules in both prostate and other solid tumors, which would further extend our knowledge to genetic defects during tumorigenesis.

## 10. Conclusions

**Figure 5:**
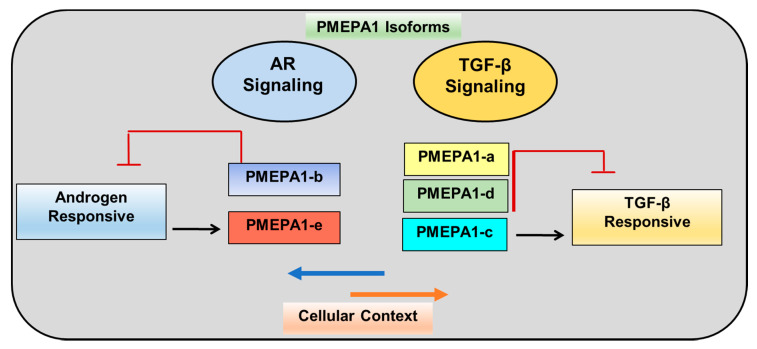
Navigation of distinct cellular signaling pathways by distinct *PMEPA1* isoform in prostate cancer context.

In conclusion, current studies confirmed the critical roles of AR and TGF-β signaling in prostate cancer development. The *PMEPA1* gene is an important regulator of AR and TGF-β signaling in prostate cancer cells. This review highlighted and summarizes the isoform specific functions of *PMEPA1* gene, stratified *PMEPA1* gene family members with expression profiling, responsiveness to androgen and TGF-β as well as cancer biological behavior in context of AR and TGF-β signaling with plausible explanations of dysfunctions of AR and TGF-β signaling in prostate tumorigenesis and aggressive progression. The roles of *PMEPA1* individual isoforms in prostate tumor initiation and progression need further clarification for undercover new prostate cancer surveillance and anti- prostate cancer therapy strategy. *PMEPA1-b* is androgen-responsive, whereas *PMEPA1-a* is TGF-β responsive and interferes with TGF-β signaling. Differential regulation of TGF-β signaling by *PMEPA1-a* and *PMEPA1-d* isoforms contributes to androgen independent stage. *PMEPA1-b* and *PMEPA1-e* are androgen responsive whereas the *PMEPA1* isoforms *a*, *c*, and *d* are TGF-β responsive and only isoforms *a* and *d* inhibited TGF-β signaling (Figure 5). Here in this review we summarized a model of prostate cancer cell adaptation from androgen dependent to hormone independent, TGF-β controlled cell growth.

## Figures and Tables

**Table 1:** Distinct *PMEPA1* motifs and phosphorylation sites.

Distinct PMEPA1 Motifs	Residues	Binding Domains	
PPPY	158–161 (C-Terminal)	WW consensus binding sequences	Xu et al., 2003 [2]
PPTY	229–232 (C-Terminal)	WW consensus binding sequences	
PPNR	186–189 (C-Terminal)	Smad	Liu et al., 2011 [18]
PPRP	112–115 (C-Terminal)	PXXP consensus binding SH3 domains	Watanabe et al., 2010 [24]
PTYP	135–138 (C-Terminal)	PXXP consensus binding SH3 domains	Giannini et al. 2003 [7]
PCPP	205–208 (C-Terminal)	PXXP consensus binding SH3 domains	
Other Predicted Motifs			
YPYL	138–141 (C-Terminal)		
YSEV	232–235 (C-Terminal)		
di-lucine	255–256 (C-Terminal)		
Other Predicted Potential Casein kinase II and Protein Kinase C Phosphorylation Site			Rae et al., 2001 [6]; Brunschwig et al., 2003 [13]
S74, S77, Y137, T217, Y219, S221, Y232, Y239 and S250			

**Table 2:** Amino acid polymorphisms reported for *PMEPA1*.

Residue Number	Polymorphism	Reference
3	SER → ARG	Peterson et al., 2010 [23] Brunschwig et al., 2003 [13]
75	TRP → ARG	
128	GLU → ASP	
179	THR → ASN	
220	SER → GLY	
228	ALA → PRO	

**Table 3:** Detection of transcripts of *PMEPA1* isoforms in prostate cells.

Cell Line	Signaling	*PMEPA1-a*	*PMEPA1-b*	*PMEPA1-c*	*PMEPA1-d*	*PMEPA1-e*
**LNCaP**	AR (+) Androgen Sensitive	(++++)	(++)	(++)	(+)	(-)
**VCaP**	AR (+) Androgen Sensitive	(+++++)	(+++)	(++)	(+)	(+)
**LAPC4**	AR (+) Androgen Sensitive	(++)	(+++)	(++)	(+)	(+)
**DU145**	AR (-) TGF-β Signaling (+)	(++)	(-)	(++)	(+)	(-)
**PC3**	AR (-) TGF-β Signaling (+)	(++)	(-)	(++)	(+)	(-)
**C4-2B**	AR (+) Androgen Independent	(+++++)	(+)	(++)	(+)	(-)
**CWR22v1**	AR (+) Androgen Independent	(++)	(+)	(++)	(+)	(-)
**BPH-1**	AR (-)	(++)	(-)	(++)	(++)	(-)
**PrEC**	AR (-)	(+++)	(-)	(++)	(+)	(-)

The color code represents: green: AR-positive (androgen sensitive); blue: AR-negative and TGF-β-signaling-positive; purple: AR-positive (androgen independent): and red: AR-negative. The numbers of (+) represents the relative transcript levels of *PMEPA1* isoforms in cells.

**Table 4:** Protein–protein interactions: cellular proteins interacting with *PMEPA1*.

Interacting Protein Partner	Domains/Motifs Involved in Binding	PMEPA1 Isoform	Reference
NEED4	PY motifs PPPY and PPTY are required to bind WW domains	*PMEPA1-b*	Xu et al., 2003 [2]
AR	Tet-Off-induced PMEPA1 protein interacts with endogenous AR protein through NEED4	*PMEPA1-b*	Li et al., 2008 [22]
Smad 2 and 3	SIM domain	*PMEPA1-a*	Watanabe et al., 2010 [24]; Liu et al., 2011 [18]
Yes-associated protein YAP65	SH3-motifs and WW-binding domains	*PMEPA1-a*	Giannini et al., 2003 [7]
GRB-2	SH3-motifs and WW-binding domains	*PMEPA1-a*	

**Table 5:** Analysis of *PMEPA1*.

Residue Number	Nature of Mutation	Functional Consequences	PMEPA1 Isoform	References
161	Y → A	Impairs interaction with NEDD4 protein	*PMEPA1-b*	Xu et al., 2003 [2]; Li et al., 2008 [22]
232	Y → A	Impairs interaction with NEDD4 protein	*PMEPA1-b*	
161 and 232	Y → A/Y → A	Impairs polyubiquitination of AR	*PMEPA1-b*	
178–181	PPNR → AAAA	Blocks nuclear translocation of Smad2 upon TGF-b stimulation	*PMEPA1-a*	Watanabe et al., 2010 [32]
>1–171 *	Deletion	Due to lack of Smad2-binding domain unable to block TGF-b receptor	*PMEPA1-a*	
>1–204 *	Deletion	Asn171- Ser204 domain is required for Smad2 interaction	*PMEPA1-a*	

* indicates that studies were carried out using *PMEPA1* derived from mouse.
